# Prospective Application of the Erythrocyte Sedimentation Rate (ESR) as a Possible Inflammatory Marker in Feline Patients

**DOI:** 10.1155/2024/2313447

**Published:** 2024-05-23

**Authors:** Eleonora Gori, Anna Pasquini, Saverio Paltrinieri, George Lubas, Carlo Militello, Daniela Diamanti, Carlo Carletti, Marianna Pantoli, Veronica Marchetti

**Affiliations:** ^1^Department of Veterinary Sciences, University of Pisa, Pisa, PI 56121, Italy; ^2^Department of Veterinary Sciences, University of Milan, Lodi, LO 26900, Italy; ^3^Clinica Veterinaria Colombo–VetPartners Italy, Lido di Camaiore, LU 55041, Italy; ^4^DIESSE Diagnostica Senese Spa, Siena, SI 53035, Italy; ^5^iVET Diagnostica Veterinaria, Flero, BS 25020, Italy

## Abstract

The application of the erythrocyte sedimentation rate (ESR) in feline medicine is currently unavailable, while in canine medicine it has been rediscovered due to the introduction of an automated ESR device. Our aims were to (1) define the reference interval (RI) of the ESR in healthy cats, (2) evaluate the ESR values between healthy and ill cats, (3) evaluate relationships between the ESR and some inflammatory markers, and (4) assess ESR changes in different durations of illness (acute, chronic, or acute-on-chronic). A prospective multicentric cohort study on 200 client-owned cats: 57 healthy cats and 143 ill cats for the other aims. Healthy cats were blood donors, or young cats underwent desexing procedures. Ill cats with full clinical medical records, hematobiochemical profiles, and diagnostic procedures to reach a final diagnosis were included. The ESR was performed with MINI-PET using the same K3-EDTA tubes used for CBC, with no additional sample required. The total leukocyte count (WBC), neutrophil-to-lymphocyte ratio (NLR), fibrinogen, serum amyloid A, and albumin/globulin ratio (A/G) were concurrently measured. Based on the clinical presentation and the final diagnosis, cats were classified as having the following: acute, chronic, and acute-on-chronic conditions. The RI of the ESR ranged between 1 and 23 mm/h. Ill cats showed a significantly higher ESR (median 29 mm/h; range 12–46 mm/h) than healthy cats (median 10 mm/h; range 1–12 mm/h; *p* < 0.0001). The ESR was positively correlated only with fibrinogen (*p* < 0.001; *r* = 0.43). Cats with acute-on-chronic diseases had the highest ESR (median 47 mm/h; range 35–56 mm/h) compared with acute (median 16 mm/h; range 14–42 mm/h; *p*=0.003) and chronic cats (median 14 mm/h; range 10–31 mm/h; *p* < 0.0001). Although further studies are needed, the ESR could be a useful ancillary inflammatory marker in cats, specifically in cats with acute diseases, with or without an underlying chronic condition.

## 1. Introduction

In physiologic conditions, glycoproteins of the red blood cell (RBC) membrane have a negative electric charge that creates a repulsive effect, called zeta potential, among cells that prevents their interactions [1]. However, during inflammation, alteration of plasma protein composition, with the proportional increase of acute phase proteins, especially fibrinogen, causes an alteration in the electric charges, and the RBCs tend to attach to each other, causing an increase in the erythrocyte sedimentation rate (ESR) [2]. In veterinary medicine, the ESR has been correlated with the mainly used acute phase proteins in dogs (i.e., C-reactive protein and fibrinogen) [3, 4]. In dogs, the ESR has been widely evaluated as an inflammatory marker as well as a monitoring parameter in various conditions, such as babesiosis, ehrlichiosis [5, 6], osteoarthritis [7], and leishmaniasis [4]. In addition, the ESR has been positively correlated with the most frequently used inflammatory proteins, such as fibrinogen and C-reactive protein [3, 4]. However, the main limitations of using the standard Westergren method for routine and clinical purposes were the high volume of blood required and the long-lasting turn-around time [8]. Recent papers investigated and validated the canine ESR measured using an automated continuous-loading instrument for the ESR [3, 9, 10], encouraging the application of the ESR in veterinary medicine. The evaluation of the ESR with MINI-PET (DIESSE, Diagnostica Senese S.p.A, Siena, Italy; https://www.diessevet.it/en/mini-pet/) is validated in dogs and horses in comparison with the gold standard Westergren method [9, 10]. The main advantages of the MINI-PET are its fast turn-around time (14 minutes to obtain the result) and the fact that no additional blood is required for the analysis since it uses the same K3-EDTA blood tubes used for the complete blood count [3, 9]. In addition, the result is processed by the instrument and displayed on the screen of the device and is not operator-dependent. The effect of time from the blood collection to the measurement and the storage temperature on the canine and feline ESR has also been recently studied [11]. This latter study demonstrated the stability of the canine ESR at room temperature for 6 hours from collection, whereas feline samples were stable for 8 hours. At refrigerated temperatures, the study highlights no difference in the T0-T24 ESR in both canine and feline samples [11]. Besides this latter study, there are no recent published papers on the feline ESR and its relationship with other inflammatory markers.

Our aims were to (1) define the ESR reference interval (RI), (2) compare ESR values between healthy and ill cats, (3) evaluate relationships between the ESR and some inflammatory markers used in feline practice, such as the total leukocyte count (WBC), neutrophil-to-lymphocyte ratio (NLR), fibrinogen, serum amyloid-A (SAA), and albumin-globulin ratio (A/G), and (4) assess ESR changes in different durations of illness (acute, chronic, or acute-on-chronic).

## 2. Materials and Methods

### 2.1. Population Selection

This multicentric prospective study was performed on left-over samples of cats presented at three veterinary facilities (the Veterinary Teaching Hospital of the University of Pisa and Milan and VetPartners Private Veterinary Clinic Colombo) between September 2022 and February 2023. For this reason, formal ethical approval was not requested. Routinely, all owners presenting at the veterinary facilities had to sign an informed consent for the scientific use of their animals' left-over blood samples. The ESR was performed on each cat presented at the three facilities and inserted into a data sheet. Then, the data sheet of raw data was revised by the authors, and the final population was composed of cats that had a full clinical evaluation with the recording of any complaints in the recent and past (months or weeks) clinical history, hematobiochemical profile, and diagnostic imaging. Clinical and diagnostic procedures were decided by the clinician in charge and were necessary to reach a final diagnosis, as it was a criterion to enroll cats in the study. In addition, cats that had a blood transfusion were excluded from the study.

### 2.2. Erythrocyte Sedimentation Rate Measurement Technique

**Figure 1:**
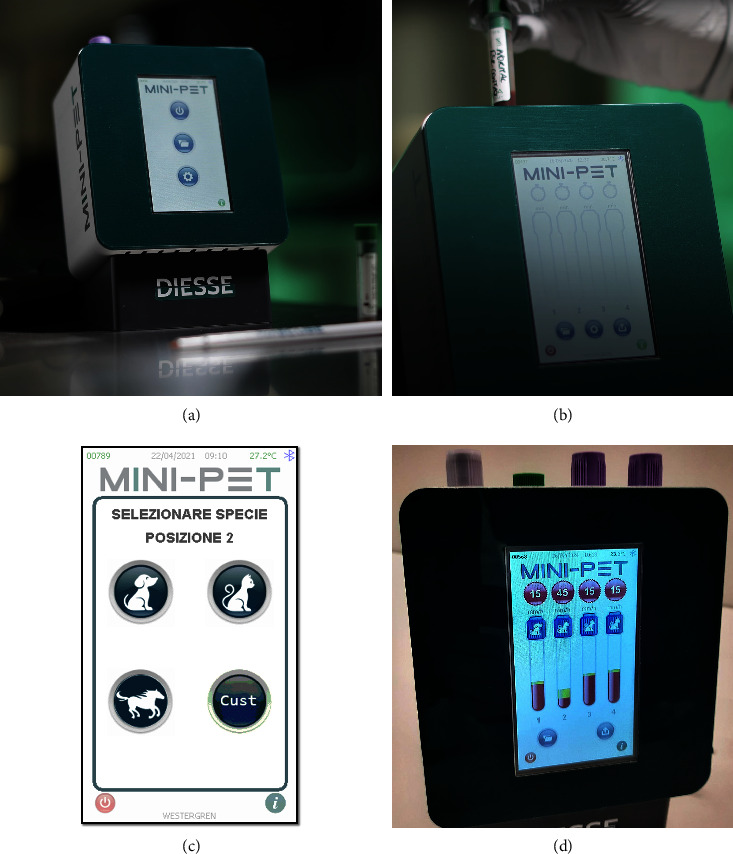
Picture representation of the use of the MINI-PET device for the ESR. (a) Appearance of MINI-PET for the ESR; (b) after the initialization, the blood tube has to be placed in one of the 4 channels of the device, and then the optical unit evaluates the level of the sample and starts the process of reading in a 14-minute countdown; (c) species selection; (d) after 14 minutes, the result is shown on the device display.

The measurement procedure of the MINI-PET device (DIESSE, Diagnostica Senese S.p.A) is reported in Figure 1 [9, 11, 12]. Briefly, the MINI-PET is an ESR point-of-care-testing machine (dimensions in mm: width 135 × height 191 × depth 125 mm) with four channels containing tube holders and optical units for the relative tubes (Figure 1(a)). After a gentle resuspension (at least ten times), the same 1-mL K3-EDTA tube immediately after the complete blood count evaluation (Idexx Procyte Dx, Idexx Laboratories, Milan, Italy, or Sysmex XN-V, Sysmex Co. Kobe, Japan) is placed in one of the four channels, and the optical units scan the tube and set the start of the 14-minute countdown for the result (Figure 1(b)). After a few seconds, the MINI-PET asks, with a message on the display, to which species the sample belongs (Figure 1(c)). After 14 minutes of the optical reading process, the ESR result (mm/h) is displayed on the device display (Figure 1(d)). If the “error” message was occurring, the K3-EDTA blood sample was removed from the device and gently resuspended 10 times, and the test was newly attempted [9, 11]. After three attempts, the sample was classified as “not measurable” and, thus, excluded from the analysis. The ESR with the MINI-PET ESR was also validated for use in cats, comparing it with the gold standard method of Westergren (described in Data S1).

### 2.3. Outline the Reference Interval of the ESR in Healthy Cats

To outline the RI for the feline ESR, a population of healthy cats was enrolled. These cats were admitted to the veterinary facilities and were defined as healthy based on their clinical history, physical examination, complete blood count, and biochemical profile. Part of these cats consisted of blood donors selected according to the Italian Ministry of Health Guidelines: age ranged from 2 to 8 years, weight 5–7 kg, regularly vaccinated, and did not receive any previous blood transfusion. In addition, each cat had to test ELISA negative for both the feline immunodeficiency virus (FIV) and the feline leukaemia virus (FeLV) [12]. Following the American Society for Veterinary Clinical Pathology (ASVCP) reference interval guidelines, due to the nonparametric distribution of the ESR in healthy cats, histograms were studied to exclude potential outliers, and Tukey's interquartile fences were used to identify multiple outliers [13]. Afterwards, a nonparametric method with a 90% confidence interval for the reference limits was applied to calculate the lower and upper reference limits [13].

### 2.4. Evaluation of the ESR between Healthy and Ill Cats and in Different Durations of Illness (Acute, Chronic, or Acute-on-Chronic)

To compare ESR values between healthy and ill cats, to correlate the ESR with the WBC, NLR, fibrinogen, SAA, and A/G, and to study results of ESR behaviour in different durations of illness, a data sheet with signalment, final diagnosis, duration of illness in ill cats (acute, chronic, or acute-on-chronic), and parameters reported earlier was built. Each cat included in the study in both the ill and healthy groups had a blood sample for their routine care, including a CBC, biochemical profile, and fibrinogen evaluation when needed. Based on the final diagnosis, clinical onset, and clinical signs duration, cats were assigned to different duration of illness groups: acute if the onset was hours to a maximum of few days and there were acute alteration in the haematological profile (e.g., CBC alteration and altered inflammatory marker, as WBC [neutrophilia/neutropenia] and/or fibrinogen, and/or SAA); chronic if the process was ongoing for weeks or months (from 3 weeks to months) and there were no signs of an active inflammatory process in the haematological profile; and acute-on-chronic if there was an acute phase (hours or days) upon a previously diagnosed chronic disease [3]. Total and differential leukocyte counts were generated using automated laser-based cell counters (Idexx Procyte Dx or Sysmex XN-V, depending on the veterinary facility), followed by a microscopic evaluation of blood smears performed by experienced clinical pathologists. The NLR was calculated based on the differential leukocyte count obtained from the microscopic blood smear evaluation. Fibrinogen (RI = 100–300 mg/dL [1–3 g/L]) was measured using an automated coagulometer (STA Compact Max Stago, FuturLab) using 1.4-ml tubes with 3.8% sodium citrate (S-Monovette®, SARSTEDT S.r.l, Milan, Italy). SAA was measured with a chemical chemistry analyzer (Beckman Coulter AU 680, Beckman Coulter s.r.l., Milan, Italy) using a commercially available turbidimetric immunoassay for human SAA (RUO LZ-SAA, Eiken Chemical Co. Ltd., Tokyo, Japan). Serum total protein and albumin were measured using an automatized biochemistry analyzer (SAT 450, Assel Srl, BT1500, Biotecnica Instruments S.p.A, Roma) using serum-plain tubes. The A/G ratio was calculated as the albumin-to-globulin ratio.

### 2.5. Statistical Analysis

The statistical analysis was performed using two statistical softwares: IBM SPSS Statistics, IBM Corp. and Reference Value Advisor [14]. The age, ESR, WBC, NLR, fibrinogen, SAA, and A/G were assessed for normality using the Kolmogorov–Smirnov test and classified as non-normally distributed if *p* < 0.05, otherwise as normally distributed variables. Non-normally distributed data were presented as the median and 25−75th percentile range, whereas normally distributed data were presented as the mean ± standard deviation (SD). Categorical variables were expressed as numbers and percentages. To define the RI of the ESR, the reference value advisor was used [13, 14]. To test any differences in the feline ESR between healthy and ill cats, the Mann–Whitney *U* test among groups was used. Correlation between the ESR and WBC, NLR, fibrinogen, SAA, and A/G was tested with Spearman's correlation test. The correlation coefficients were interpreted as follows: 0.1–0.3 weak correlation, 0.4–0.6 moderate correlation, and 0.7–0.9 strong correlation [15]. The presence of the abnormal ESR was also compared among cats with or without abnormal WBC count (abnormal if WBC< 2,000/*μ*L [<2 × 10^9^/L] or >17,000/*μ*L [>17 × 10^9^/L] and/or the presence of band neutrophils) and with or without abnormal NLR (NLR>4.5) [16], fibrinogen (>300 mg/dL [>3 g/L]), and SAA (>3 mg/L) using the Chi-squared test. Finally, the ESR was also compared among disease groups (acute, chronic, and acute-on-chronic) using the Kruskal–Wallis test with Wilcoxon pairwise comparisons with the Bonferroni correction test. A *p* value <0.05 was considered statistically significant.

## 3. Results

The study included left-over blood samples obtained from a feline population composed of 200 cats: 143 ill and 57 healthy cats. Demographics and clinical data about the study population are reported in Table 1. Healthy cats were significantly younger than ill cats (*p* < 0.001). During ESR measurements, a total of 114 error messages occurred on 41 samples. Eighty-six error messages have been resolved using the procedure described in the methods. All these 114 error messages occurred in the ill cats group.

**Figure 2:**
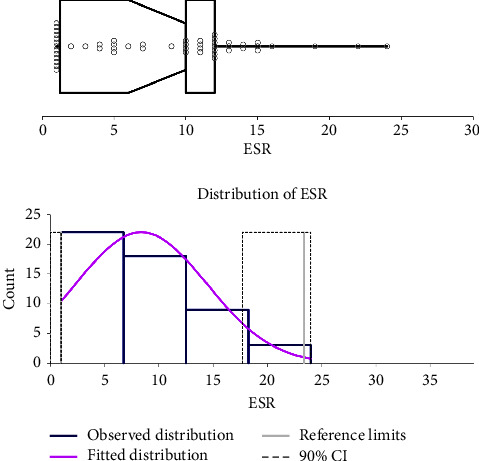
Histogram and graph regarding the erythrocyte sedimentation rate in a population of 52 healthy cats.

Following the ASVCP guidelines for the RI, the healthy population was used to calculate the RI (*n* = 57). After the elimination of outliers (*n* = 5) (cats used for RI = 52), the ESR RI ranged (minimum-maximum range) between 1 and 23 mm/h (90% CI for lower limit 1-1 mm/h and 90% CI for upper limit 17–24 mm/h). The histogram and graph regarding the RI are reported in Figure 2.

**Figure 3:**
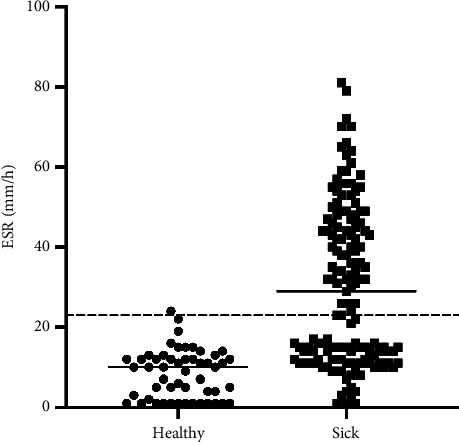
Scatterplot of erythrocyte sedimentation rate (ESR) values in healthy (dots) and ill cats (squares) (*p* < 0.001). The dotted line represents the ESR upper reference interval (1–23 mm/h), and the plain horizontal line within the scatterplots represents the median of the ESR in each group.

Ill cats showed a significantly higher ESR (median 29 mm/h; range 12–46 mm/h) compared with healthy cats (median 10 mm/h; range 1–12 mm/h; *p* < 0.0001; Figure 3).

**Figure 4:**
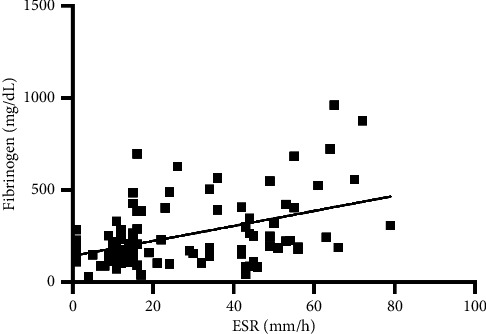
Correlation between the ESR and fibrinogen (*p* < 0.001*r* = 0.4).

In Table 2, the correlations between the ESR and the other inflammatory markers are reported. Briefly, no significant correlations were found between the ESR and WBC, NLR, SAA, or A/G. Conversely, a significant but weak correlation was found with fibrinogen (Figure 4). In addition, the Chi-square analysis demonstrated that the proportion of cats with an abnormal ESR (70%) was significantly higher in cats with abnormal fibrinogen (*p*=0.002). In addition, another association between cats with an abnormal ESR (>23 mm/h) and an abnormal NLR (>4.5) was found (*p*=0.004). No association between the abnormal ESR and abnormal WBC, SAA, or A/G was found (all *p* > 0.05).

**Figure 5:**
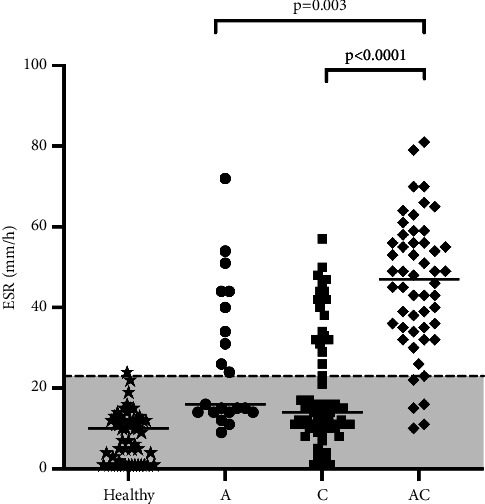
Scatterplot of erythrocyte sedimentation rate (ESR) values in healthy, acute (A), chronic (C), and acute-on-chronic (AC) disease groups. Horizontal lines within the four scatterplots represent the median value. The gray band represents the reference interval for the feline ESR determined as described earlier.

Based on the duration of illness, cats were divided into chronic (*n* = 70), acute-on-chronic (*n* = 52), and acute diseases (*n* = 21). Cats with acute-on-chronic diseases had the highest ESR (median 47 mm/h; range 35–56 mm/h) compared with acute (median 16 mm/h; range 14–42 mm/h; *p*=0.003) and chronic cats (median 14 mm/h; range 10−31 mm/h; *p* < 0.0001). The ESR of acute and chronic cats did not differ significantly from each other (*p*=0.18; Figure 5).

## 4. Discussions

Our study highlighted the prospective use of the ESR in feline medicine, which is considered a cheap and easy method to measure this parameter. It has also proved to be a reliable marker of inflammation in cats, showing a moderately positive correlation with fibrinogen. In our study, ill cats with inflammatory diseases showed a higher ESR compared with healthy cats or cats with chronic conditions.

Even if the reference method for measurement of the ESR is still the Westergren method [8], an optical veterinary instrument, the MINI-PET, has been studied and validated in dogs, horses [3, 9–11], and now in cats. Unfortunately, when the Westergren method was used, the measurement of the ESR took approximately 1 hour, and the reading of the tube, and thus the ESR result, was operator-dependent [8]. Prior to this study, the MINI-PET was validated for veterinary ESR evaluation in dogs [9] and horses [10] using the Westergren method as the gold standard. In both dogs and horses, the ESR with MINI-PET correlated well with the Westergren method (*r* coefficient of 0.76 for dogs and 0.86 for horses) [9, 10]. We perform the validation of the ESR with MINI-PET using 63 cats in comparison with the gold standard Westergren method (refer to Data S1). These results showed an intra- and interassay CV of 0.04 and 0.49, respectively, an excellent correlation with the gold standard method (*r* = 0.86), and the lack of proportional or constant errors.

The MINI-PET company provided a RI for the MINI-PET ESR of 1–11 mm/h, which was based on internal company reports and studies. However, an official RI for the feline ESR has not been published yet; as a primary aim, we wanted to define the RI for the feline ESR using the new ESR machine firmware release with the results after 14 minutes instead of after 20 minutes [9]. We decided to include not only cats from the blood donor program, which must fit the precise criteria established by the Healthcare Ministry, but also young cats presented for desexing procedures such as ovariectomy and orchiectomy to increase the RI sample population. The newly established RI for the ESR resulted in 1–23 mm/h. This RI was reached first by eliminating outliers and applying a nonparametric method, as stated in ASVCP guidelines [13]. Each eliminated outlier belonged to an intact female, and at least 3 of them were in oestrus at the time of the ESR measurement. It must be specified that we define the RI for the ESR without taking into account the signalment characteristics (age, sex, and/or sex status) but only the healthy status. For future perspectives, it would be interesting to study the ESR in different age ranges, sexes, and intact vs. spayed cats.

The ESR showed a moderately positive correlation with fibrinogen, and it was not correlated with any other inflammatory marker considered. This finding was also supported in two recent studies performed on dogs [3, 4]. In cats, fibrinogen is a minor acute-phase protein, although it is associated with inflammation [17, 18]. In healthy animals, the plasma fibrinogen level is constant, but when an inflammatory condition is acute, hepatocytes may release large amounts of fibrinogen [17]. However, fibrinogen has not been studied in relation to the ESR in cats. It is well known that the ESR is mainly determined by the balance between pro-sedimentation, especially fibrinogen, and anti-sedimentation factors, especially haematocrit. However, the low correlation coefficients may be due to the minor role of fibrinogen in feline inflammatory processes [19].

However, despite the interesting results reported earlier, no association or correlation between the ESR and SAA was found. SAA is considered one of the most representative inflammatory markers in cats [20]. The behaviour of SAA has been investigated in cats that underwent surgery procedures, and, unrelated to the type of surgery, SAA increased rapidly and returned to normality within five days [21, 22]. However, even if the kinetics of SAA are well known, the magnitude of the increase and its clinical utility have involved many studies with different results depending on the underlying condition [19]. In relation to its fast decrease, SAA may not be as useful in chronic conditions or in mild inflammatory diseases [20, 23]. In addition, a possible bias in our results may be represented by the methodology of measurement of SAA. In fact, in our study, SAA was measured using a turbidimetric assay with antihuman-SAA-specific antibodies validated in different cats (LZ-SAA), demonstrating an acceptable imprecision, and there is no sign of significant inaccuracy [24]. The use of the previous assay and the use of frozen-stored samples for SAA measurement may have influenced our results since only data about canine SAA stability are available (stable for 6 months frozen at −20°C and for 5 days refrigerated) [25]. In addition, both different onsets and half-life of SAA and the behaviour of the ESR may influence our results, especially in acute-on-chronic diseases. The SAA is comparable with the CRP assay in humans and dogs for detecting inflammation and takes into account the differences between species in the production of acute-phase proteins. In human medicine, it is already well stated that the CRP and ESR measurements did not overlap in detecting an inflammatory process, and the disagreement between these two tests has been evaluated in several papers [26–29]. So far, in dogs, the correlation between CRP and ESR is similar to that in humans, confirming the lack of a strong correlation [3]. So, the lack of correlation of the ESR with SAA obtained in this study in cats could be a comparable effect shown in humans and dogs for the CRP. Indeed, SAA, similarly to CRP, is a fast APP that decreases in few days, while fibrinogen has a delayed peak and has a prolonged decrease after the onset of an inflammation. In addition, based on the different kinetics of SAA, fibrinogen, and ESR, the evaluation of the ESR might be useful, especially in acute-on-chronic diseases.

We also wanted to test the potential association and correlation between the ESR and NLR. Specifically, we found that approximately 60% of cats with an abnormal ESR also had an NLR > 4.5. In fact, the utility of the feline NLR has been investigated to discriminate between feline SIRS and sepsis in healthy cats and proved to be a good discriminating marker between SIRS/septic cats and healthy cats with a cut-off value of 4.5 [16]. In addition, the NLR seems to be related to mortality in SIRS and septic cats, proving that it may be a useful tool in these patients [16]. Based on this consideration and because NLR may be influenced by a wide variety of magnitudes, further investigations are needed to understand the real clinical significance of the ESR-NLR relationship.

Lastly, the ESR was more frequently abnormal in cats with acute-on-chronic diseases compared with acute and chronic ones and thus more useful in those specific disease presentations. We wanted to evaluate the ESR in different settings of disease (acute, chronic, and acute-on-chronic), as has been performed on dogs [3], allowing us to understand that the ESR finds its best analytical performance in feline acute-on-chronic diseases. The lack of active inflammation in chronic conditions may be the cause of this specific finding [20]. Also, in acute-on-chronic diseases, the primary chronic condition may have affected the acute inflammatory response. Especially because, in this type of patient, there is a subclinical acute phase reaction, and the cells involved in the innate immune response constantly produce and release proinflammatory cytokines [30]. This mechanism may contribute to our result, in which cats with acute-on-chronic disease had the highest ESR values.

This study has some limitations. The RI of the ESR has been established on 57 cats, and after the elimination of outliers, the final healthy population for the RI was composed of 52 cats. It would be obviously interesting and needed to increase the healthy population to refine and confirm the RI of the feline ESR. Each inflammatory marker was not available for all cats. Based on a previous study [3, 9], there were no performance differences in the ESR between anaemic and nonanaemic animals. We did not investigate the relationship with anaemia, especially because it would require additional time and adjustments based on haematocrit. However, it may be interesting to increase the population to evaluate more factors such as haematocrit and the mean corpuscular volume of the feline ESR. Especially in cats, some ESRs assessed by the Westergren method may be biphasic, that is, a clear line or separation between RBCs and plasma is not observed in the sample tube (i.e., severe hemolysis, auto-agglutination). In those cases, the MINI-PET is likely to display an error message [9]. Lastly, variables such as age, sex, and breed were not considered in this study, so their influence on the ESR remains unknown.

## 5. Conclusions

The MINI-PET has several advantages: no additional blood required for the analysis (extremely important in cats; uses the same tube utilized for the complete blood count), rapid turn-around time (14 minutes), not operator-dependent reading (automated optical unit), and, thus, more objective analytical data. On the contrary, the MINI-PET showed some difficulties (error messages) when analysing the diphasic ESR or samples with microagglutination, and the price of the device is higher than the Westergren equipment (Westergren tubes). The evaluation of the ESR in feline medicine may be a realizable tool in the inflammation assessment, especially because when using the MINI-PET, no additional blood is required for the analysis. Moreover, since the ESR does not show a high correlation with any inflammatory markers analysed in this study, it could be studied in specific inflammatory diseases to explore its potentiality as a novel marker to monitor feline health status. In addition, the ESR seemed to be more frequently abnormal in cats with acute-on-chronic diseases compared with acute and chronic ones and thus more useful in acute-on-chronic disease presentation.

## Figures and Tables

**Table 1:** Demographics and clinical data about the study population.

Variables	Overall population	

Animals	200 cats	Healthy ➜ 57 cats
		Ill ➜ 143 cats

Age	Median age 5.6 years (range 2.1–10.9 years)	Healthy ➜ 3 years (range 1.2–5.3 years)
		Sick ➜ 8 years (range 3.2–11.9 years)

Sex	90♀	Healthy ➜ 34♂ (21 castrated + 13 intact)
		23♀ (13 spayed + 10 intact)
	110♂	Ill ➜ 76♂ (54 castrated + 22 intact)
		67♀ (54 spayed + 13 intact)

Breed	158 cats = domestic short hair	Healthy ➜ domestic short hair (*n* = 45), Maine Coon (*n* = 8), British short hair (*n* = 2), chartreux (*n* = 1), and Birman (*n* = 1)
	42 cats = pure breed	Ill ➜ domestic short hair (*n* = 133), Birman (*n* = 2), Siamese (*n* = 2), Angora, British short hair, Maine coon, Norwegian Forest, Persian, and Siberian cat (1 cat for each breed)

**Table 2:** Correlation between the ESR and other inflammatory markers in the study population.

	Fibrinogen *n* = 109	WBC *n* = 200	NLR *n* = 200	SAA *n* = 107	A/G *n* = 182
Correlation coefficient (*r*)	0.4	0.01	0.12	0.09	0.03
*p* value	<0.001	0.9	0.08	0.35	0.64

WBC, white blood cell count; NLR, neutrophil-to-lymphocyte ratio; SAA, serum amyloid-A; A/G, albumin-to-globulin ratio.

## Data Availability

The datasets presented in this article are not readily available because they are properties of DIESSE Diagnostica Senese Spa. Requests to access the datasets should be directed to DIESSE Diagnostica Senese Spa.

## Supplementary Materials

Supplementary Materials Data S1: validation study for MINI-PET ESR.
