# Supplementary material for: Prospective Application of the Erythrocyte Sedimentation Rate (ESR) as a Possible Inflammatory Marker in Feline Patients
Source: Vet Med Int. 2024 May 23;2024:2313447. doi: 10.1155/2024/2313447 (PMC11139534; doi:10.1155/2024/2313447)
Supplement: Supplementary Materials — Data S1: validation study for MINI-PET ESR. [file 2313447.f1.docx]

Data S1: Validation study for ESR using MINI-PET.

Sixty feline blood samples from both sick and healthy cats were used for the validation study. For each cat, the ESR was determined both with the MINI-PET (ESR-MINIPET) and the Westergren method (ESR-W) within 2 and 4 hours from sampling. The determination of the ESR- MINIPET involves the use of the MINI-PET instrument (DIESSE, Diagnostica Senese S.p.A., Italy), and the procedure of measurement is described in the manuscript. ESR-W was measured following the International Council for Standardization in Haematology (ICSH) guidelines [Jou et al. 2011]. To investigate and evaluate the ESR-MINIPET method, the following statistical work was performed: intra-assay and inter-assay coefficient of variation (CV), correlation (*r*) between ESR-W and ESR-MINIPET values, R^2^ coefficient, and Bland-Altman test. All these tests were performed using MedCalc® statistical software.

Intra- and inter-assay CVs were 0.04% and 0.49%, respectively. The correlation coefficient (*r*) between ESR-MINIPET and ESR-W was 0.85 (95% CI 0.76-0.92). Based on the regression analysis, the ESR-MINIPET appears linear without significant deviation (R^2^=0.74). As reported in the Bland-Altman plot (Figure 1), values measured by ESR-MINIPET agreed with those measured with ESR-W with a mean difference of 0.1 with a lower limit of -21.8 and an upper limit of 21.6.


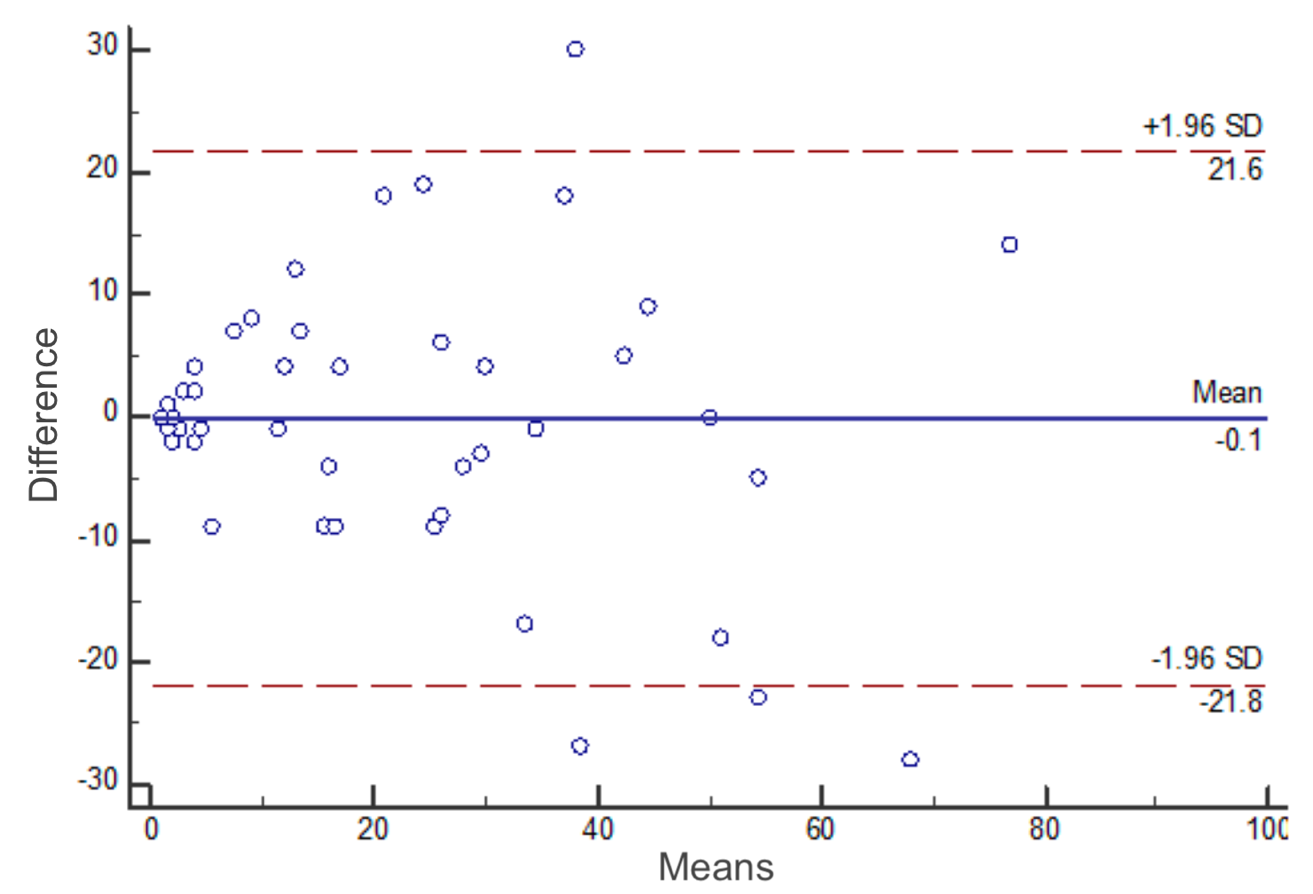


**Figure 1**. Bland-Altman plot of ESR-MINIPET and ESR-W.
